# Bovine Lactoferrin Promotes Neurite Outgrowth in PC12 Cells via the TrkA Receptor

**DOI:** 10.3390/ijms252011249

**Published:** 2024-10-19

**Authors:** Daichi Nagashima, Noa Mizukami, Nana Ogawa, Sayaka Suzuki, Megumi Ohno, Ryoken Aoki, Megumi Furukawa, Nobuo Izumo

**Affiliations:** 1 Laboratory of Clinical Pharmaceutics, Yokohama University of Pharmacy, Yokohama 245-0066, Kanagawa, Japan; daichi.nagashima@yok.hamayaku.ac.jp; 2 General Health Medical Research Center, Yokohama University of Pharmacy, Yokohama 245-0066, Kanagawa, Japan; 3 Laboratory of Pharmacotherapy, Yokohama University of Pharmacy, Yokohama 245-0066, Kanagawa, Japan; 4 NRL Pharma Inc., Kawasaki 213-0012, Kanagawa, Japan; 5 Center for Pharmaceutical Education, Yokohama University of Pharmacy, Yokohama 245-0066, Kanagawa, Japan

**Keywords:** lactoferrin, PC12 cells, p44/42, TrkA receptor, neurite outgrowth

## Abstract

Lactoferrin (LF) is a multifunctional protein abundant in breast milk that modulates the functions of neural stem cells. Recent studies have demonstrated the efficacy of bovine LF (bLF) in mitigating behavioral changes; however, the molecular mechanisms on the nervous system have not yet been elucidated. The presented study aimed to characterize the molecular mechanisms of bLF on nerve extension in PC12 cells. PC12 cells were treated with 0.01–1000 µg/mL of bLF, and cell viability was determined using the cell counting kit-8 assay after treatment for 24 h. Morphometric evaluation was performed after 24 or 72 h of treatment with 50 ng/mL nerve growth factor (NGF) or 100–500 µg/mL bLF. The molecular mechanisms were investigated using Western blotting and real-time quantitative PCR. Cell viability was significantly decreased after treatment with 600–1000 µg/mL bLF for 24 h compared with the control group. Morphometric evaluation revealed neurite outgrowth after 72 h of NGF treatment, with a significant increase in neurite outgrowth after treatment with 250 µg/mL bLF. The phosphorylated p44/42 expression ratio peaked at 5 min and persisted for up to 10 min. Quantitative real-time PCR revealed a significant decrease in MAP2 expression. Our findings suggested that bLF enhanced PC12 cell neurite outgrowth to a similar extent as NGF. These effects are thought to be mediated via the TrkA receptor and activated by the phosphorylated ERK signaling pathway. Therefore, this study demonstrates that bLF promotes neurite outgrowth via a pathway similar to that of NGF.

## 1. Introduction

Lactoferrin (LF) is an iron-binding glycoprotein with a molecular weight of 83,000 Da that was first identified in milk by Sorensen et al. in 1939 [1]. LF is particularly abundant in the colostrum (6–8 mg/mL), playing an essential role in preventing infections in neonates [2]. In its apo-type state (free iron ions present), LF binds to Fe^3+^ and/or Cu^2+^, thereby protecting cells and genes from oxidative damage [3]. It is also present in saliva, tears, bile, pancreatic juice, and neutrophils in adults, and its pharmacological effects include infection prevention, anti-inflammation, anti-allergic, bone metabolism, anticancer, enhancing lipid metabolism, and pain and anxiety relief [4,5,6]. Owing to its multifunctionality and safety in the body, as it is naturally present and ingested in large quantities by infants through breast milk, LF-containing functional health foods and supplements have recently been marketed, with limited adverse events reported. The bovine-derived LF (bLF) used in this study is a food additive deemed safe by the US Food and Drug Administration (FDA) [7]. Thus, the safety of LF is considered established. Moreover, it is reported that bLF induced the nerve growth factor (NGF) synthesis in mouse L-M cells [8].

NGF is a protein isolated from snake toxin and the submaxillary glands of male mice that functions as a neurotrophic factor and supports the survival of developing sympathetic and cutaneous sensory neurons [9,10,11]. Both high-affinity (TrkA) and low-affinity NGF (p75) receptors exist in the body. When NGF is absorbed through the TrkA receptor, it maintains survival and neurite outgrowth and promotes neurotransmitter synthesis in nerve cells [12,13]. In contrast, the p75 receptor enhances TrkA receptor activity and induces apoptosis [14,15]. NGF binds to the TrkA receptor on the cell surface and activates the serine/threonine kinase Raf via the small G-protein Ras [13]. Subsequently, Raf phosphorylates mitogen-activated protein kinase (MAPK)/ERK kinase (MEK), and phosphorylated MEK phosphorylates p44/42 (extracellular signal-regulated kinase, ERK), leading to the activation of the ERK pathway [16]. Consequently, various cellular responses occur, such as the phosphorylation of various proteins in the cytoplasm and nucleus and the induction of transcription regulatory factors [17]. Additionally, NGF specifically targets peripheral sympathetic nerve cells, sensory nerve cells, cholinergic neurons in the basal forebrain of the central nervous system, and striatal cholinergic neurons [18]. Among these, TrkA receptor expression is associated with cholinergic neurons in the basal forebrain, suggesting a noteworthy relationship with cognitive function and learning ability [19,20].

Our previous study reported that bLF effectively recovered behavior in an ovariectomized mouse model of depression [21]. However, the mechanisms underlying this process are yet to be clarified in the nervous system. This study aimed to characterize the pharmacological effects of bLF using cell viability assays, morphological analysis, and protein expression evaluation to investigate the molecular mechanisms of neurite outgrowth.

## 2. Results

### 2.1. Evaluation of Cell Viability

**Figure 1:**
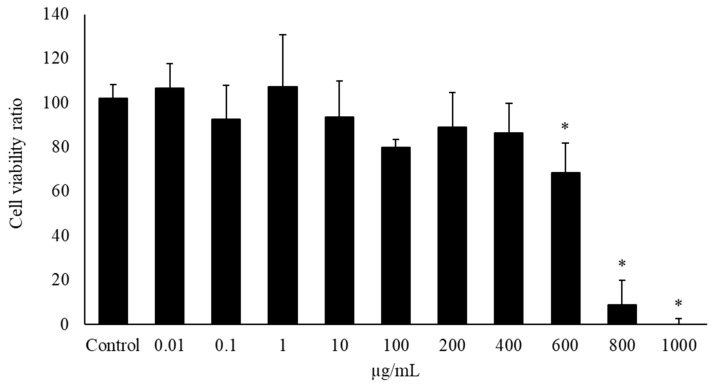
Cell viability ratio of bovine lactoferrin (bLF) using the cell counting kit-8 (CCK-8) assay. PC12 cells were seeded onto 96-well plate and treated to bLF at concentrations ranging from 0 to 1000 μg/mL for 24 h. After treatment, CCK-8 reagent was added before incubation for 1 h. Absorbance was measured at 450 nm on an automated plate reader. Data values are presented as the mean ± standard deviation (SD). * *p* < 0.05, compared with the control.

Appropriate bLF concentrations for the administration to PC12 cells were determined using the CCK-8 assay to evaluate bLF cytotoxicity. Treatment of PC12 cells with 0.001–1000 µg/mL bLF for 24 h exhibited no effects until 400 µg/mL; however, treatment with 600, 800, and 1000 µg/mL bLF for 24 h significantly decreased cell viability compared with the control groups (Figure 1). In the subsequent experiments, doses of less than 600 µg/mL bLF were used.

### 2.2. Morphological Evaluation of bLF

**Figure 2:**
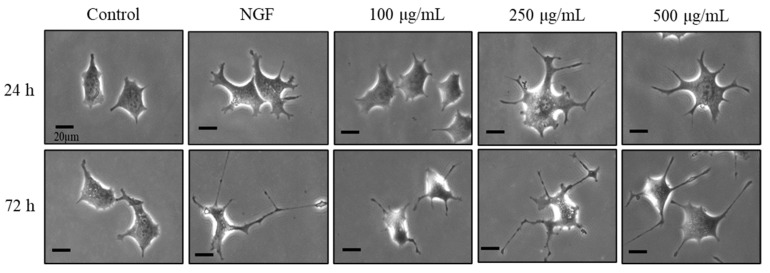
Representative PC12 cell images at 24 and 72 h after treatment with 100, 250, and 500 µg/mL bLF or 50 ng/mL NGF. Scalebar = 20 μm. NGF, nerve growth factor.

**Figure 3:**
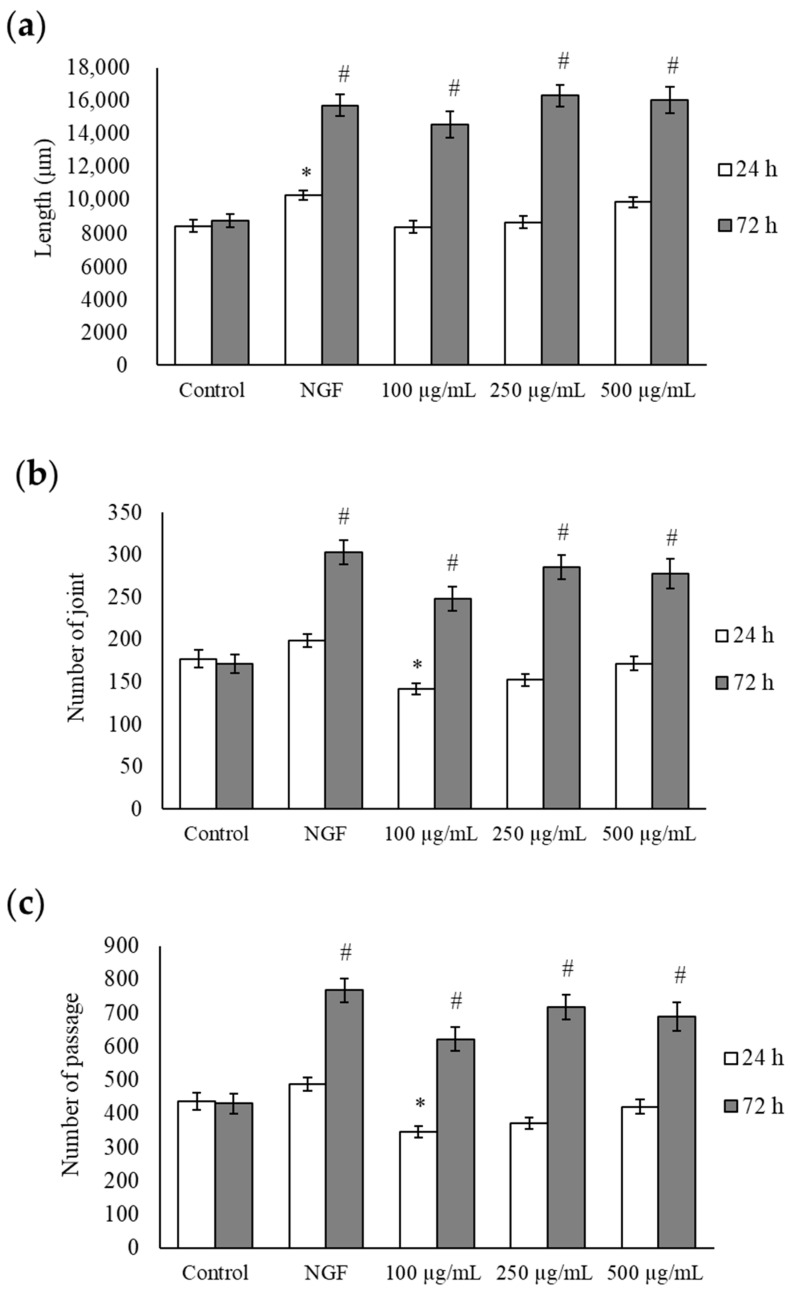
Morphological evaluation, including length (**a**), joints (**b**), and passages (**c**), at 24 and 72 h after treatment with each substance. Data values are presented as the mean ± SD. * *p* < 0.05, compared with the 24 h control group; # *p* < 0.05, compared with the 72 h control group. NGF, nerve growth factor.

**Figure 4:**
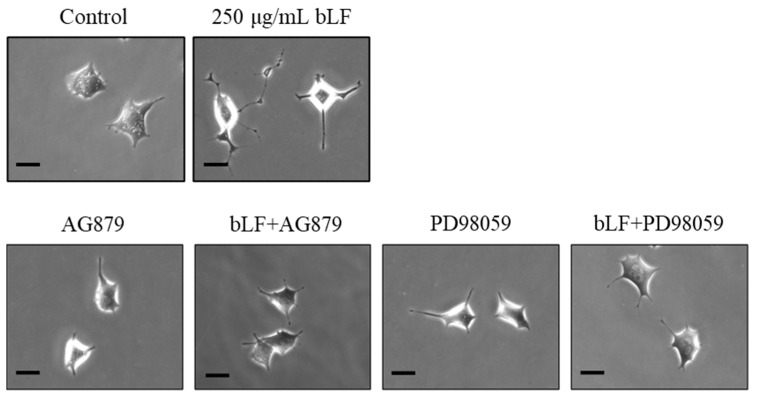
Representative PC12 cell images 72 h after individual administration of 250 µg/mL bLF, 10 µM AG879, and 10 µM PD98059, or simultaneous administration of 250 µg/mL bLF and 10 µM AG879 or 10 µM PD98059. Scalebar = 20 μm. bLF, bovine lactoferrin; AG, AG879; PD, PD98059.

**Figure 5:**
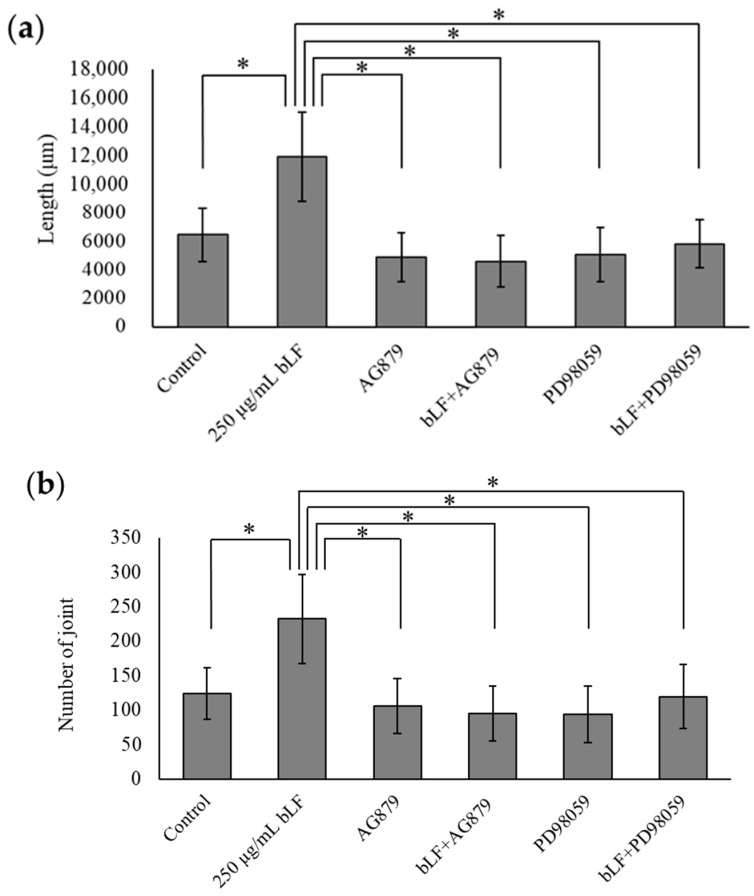

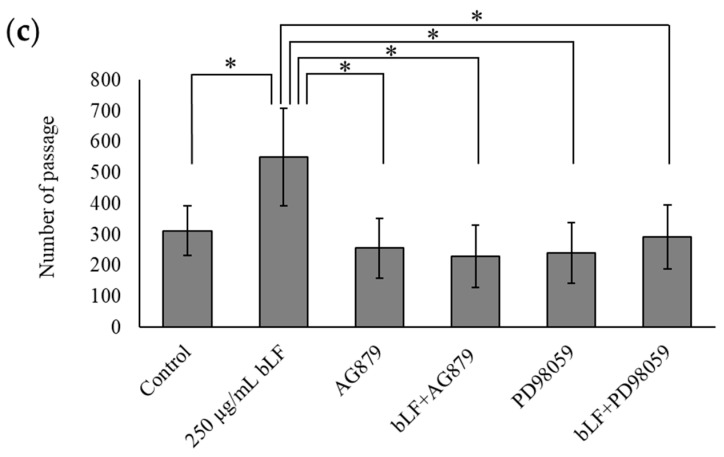
Morphological evaluation, including length (**a**), joints (**b**), and passages (**c**), at 72 h after treatment with each substance. Data values are presented as the mean ± SD. * *p* < 0.05. bLF, bovine lactoferrin.

Changes in neurite outgrowth length, joints, and passages at 24 h are shown in Figure 2 and Figure 3a–c. A single administration of NGF for 24 h significantly increased the length, joints, and passages compared to the control group. A single administration of NGF or 100, 250, and 500 µg/mL bLF for 72 h significantly increased the length, joints, and passages compared to those in the control group. Conversely, simultaneous administration of 250 µg/mL bLF and 10 µM AG879 or PD98059 for 72 h significantly decreased the length, joints, and passages compared to a single administration of bLF (Figure 4 and Figure 5a–c). Moreover, single administration of 10 µM AG879 or PD98059 for 72 h did not induce neurite outgrowth in PC12 cells.

### 2.3. Phosphorylated p44/42 Expression Levels

**Figure 6:**
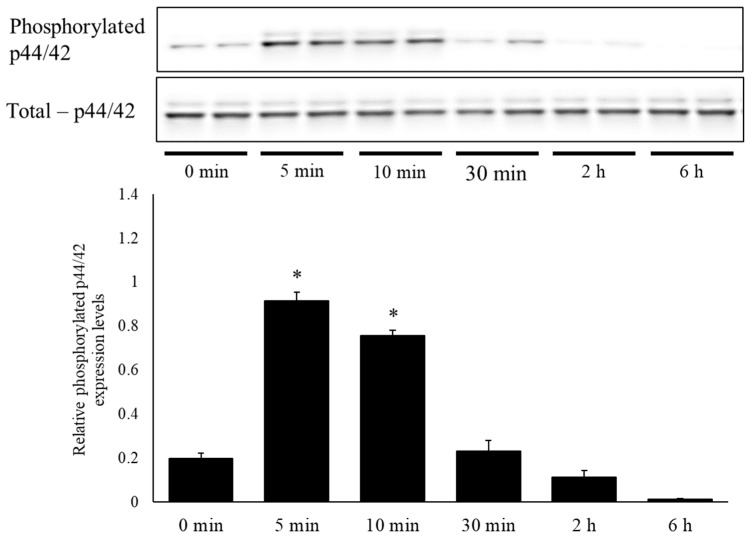
Time-related changes in phosphorylated p44/42 and total p44/42 levels in PC12 cells and the ratio of relative quantities. Changes in the relative protein expression levels of phosphorylated p44/42 and the total p44/42 ratio were analyzed via Western blot analysis. The relative protein levels were expressed as the mean ± SD. * *p* < 0.05, compared with the 0 min group.

**Figure 7:**
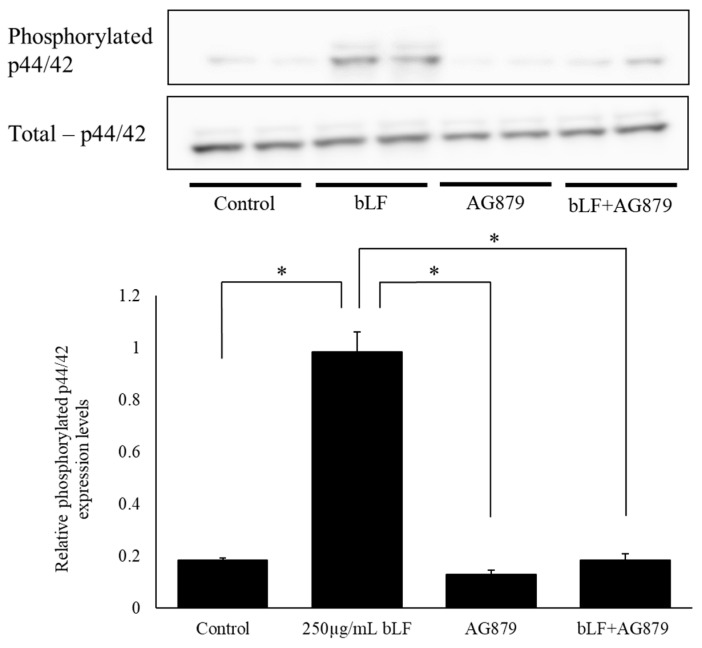
Effect of TrkA receptor inhibitor on phosphorylated p44/42 and total p44/42 levels in PC12 cells, measured using Western blot analysis, and the ratio of relative quantities. Changes in the relative protein expression levels of phosphorylated p44/42 and the total p44/42 ratio were analyzed via Western blot analysis. The relative protein levels were expressed as the mean ± SD. * *p* < 0.05.

**Figure 8:**
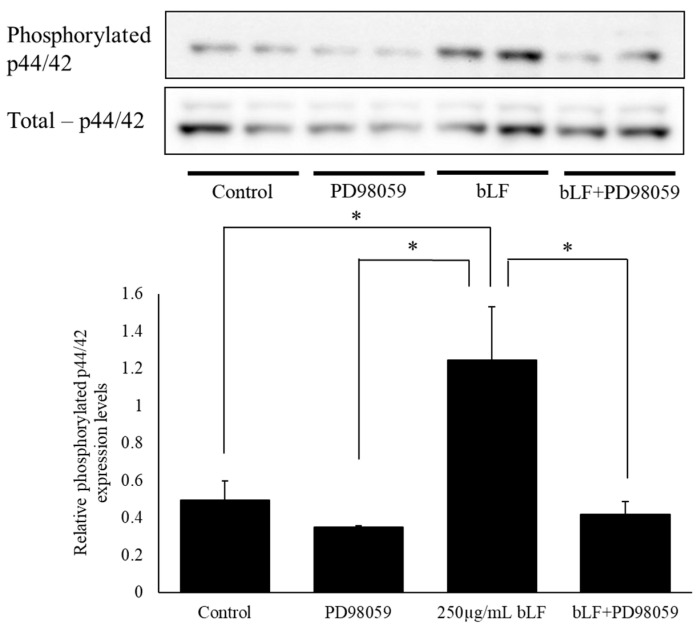
Effect of the selective MEK inhibitor on phosphorylated p44/42 and total p44/42 levels in PC12 cells, measured using Western blot analysis, and the ratio of relative quantities. Changes in the relative protein expression levels of phosphorylated p44/42 and the total p44/42 ratio were investigated via Western blot analysis. The relative protein levels were expressed as the mean ± SD. * *p* < 0.05.

To investigate the mechanisms of bLF in neurite outgrowth, we performed Western blot analysis to measure total p44/42 and phosphorylated p44/42 protein levels. The results showed that the ratio of phosphorylated p44/42 expression levels was significantly increased following a single administration of 250 µg/mL bLF for 5 and 10 min compared to the levels at 0 min. However, there was no significant change in the ratio of phosphorylated p44/42 expression following 30 min, 2 h, or 6 h of treatment (Figure 6). In contrast, using a TrkA receptor inhibitor (AG879) along with a selective MEK inhibitor (PD98059) significantly decreased the ratio of phosphorylated p44/42 expression 5 min after bLF treatment (Figure 7 and Figure 8).

### 2.4. Synapsin-1, Synaptophysin, and MAP2 Expression Levels Measured Using RT-qPCR

**Figure 9:**
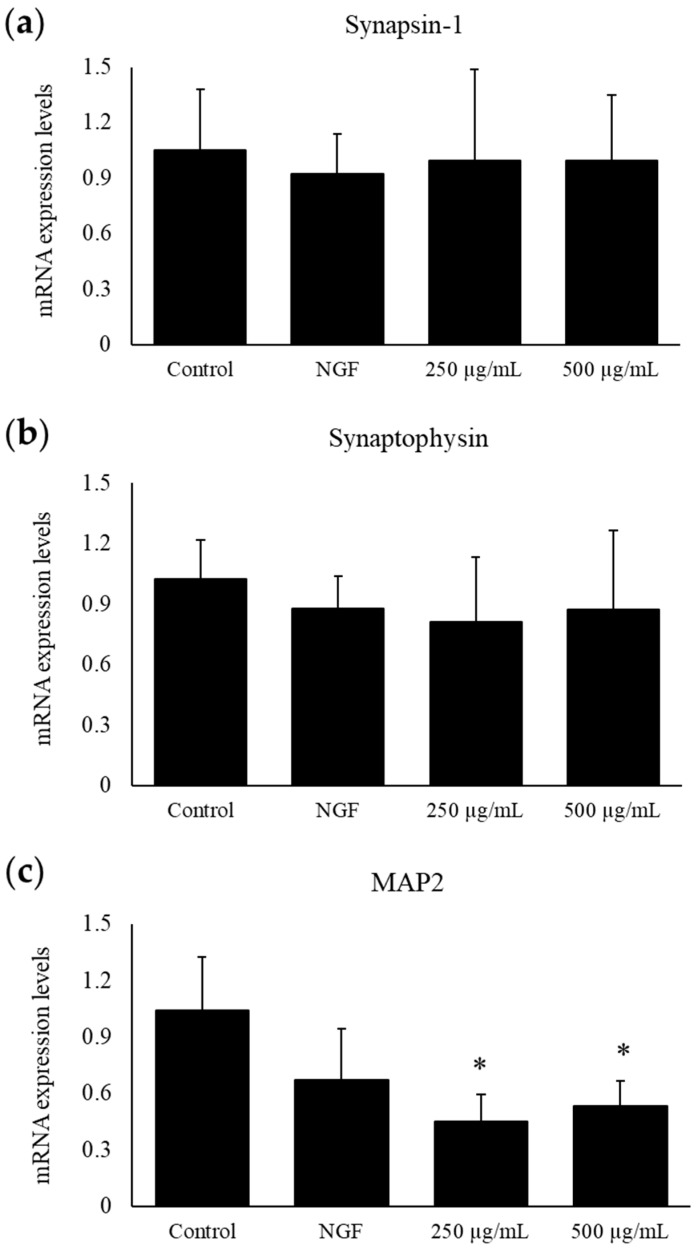
The mRNA expression levels of synapse-related markers, synapsin-1 (**a**), synaptophysin (**b**), and MAP2 (**c**), after treatment with 250 or 500 µg/mL bLF for 24 h, measured using real-time quantitative PCR. Total RNA was isolated 24 h after bLF treatment. Data are presented as the mean ± SD; * *p* < 0.05, compared with the control. NGF, nerve growth factor.

After incubation with bLF for 24 h, mRNA was isolated to determine the expression of synapsin-1, synaptophysin, and MAP2. The synapsin-1 and synaptophysin levels did not change compared with those of the control; however, the MAP2 expression levels decreased significantly from those of the control after treatment with 250 or 500 μg/mL bLF (Figure 9a–c).

## 3. Discussion

The primary findings of this study were that the administration of NGF and bLF induced the following changes in PC12 cells: (1) bLF could affect cytotoxicity at concentrations above 600 µg/mL but did not affect cytotoxicity at concentrations of 0.01–400 µg/mL; (2) treatment with 100, 250, and 500 µg/mL bLF for 72 h significantly increased neurite outgrowth; (3) neurite outgrowth induced by NGF and bLF was suppressed by 10 µM AG879 and 10 µM PD98059; (4) neurite outgrowth mediated by bLF was accompanied by an increase in phosphorylated ERK expression after 5 min, which was suppressed by AG879 and PD98059; and (5) neurite outgrowth was altered by a decrease in MAP2 mRNA expression levels.

Our previous study reported that LF exerted antidepressant effects on behaviors that were suppressed in ovariectomized mice. However, the molecular mechanisms underlying the effects of LF on nerve cells have not yet been elucidated. The present study investigated the effects of LF treatment on neurite outgrowth, focusing on morphological assessment and employing various inhibitors to elucidate the underlying mechanisms. PC12 cells are commonly employed for neuropharmacological evaluations; they extend neurites and differentiate into neuron-like cells in response to NGF stimulation [22]. To investigate neurite outgrowth, morphological evaluation was performed using NGF as a positive control. Morphological evaluation showed that neurite outgrowth in the bLF-treated group was significantly increased after 72 h and was comparable to that under NGF stimulation. Furthermore, this study revealed that the optimal LF concentration for neurite outgrowth was 250 µg/mL, and this effect was observed 72 h after treatment with bLF alone. bLF contains approximately 10% iron [23], and the effect of iron on neurite outgrowth has been previously reported [24]. Therefore, the iron in bLF is suggested to play a role in enhancing neurite outgrowth mediated by bLF; however, the exact amount of iron contained in the bLF used in the present study is unknown. It has also been reported that bLF induces the synthesis of NGF in mouse fibroblast-like L-M cells; however, the observed induction of NGF production could not be attributed to the stimulation of iron transport [8]. Further investigations are needed to understand the physical properties of bLF and corroborate that its pharmaceutical effects are related to iron.

The activation of the MAPK pathway is necessary for NGF-induced neurite outgrowth [25]. The MAPK signaling pathway transmits signals to the nucleus via the Ras-Raf-MEK-ERK cascade [26]. Western blot analysis revealed a significant increase in the ratio of phosphorylated ERK to total ERK following treatment with 250 µg/mL bLF for 5 and 10 min compared to the 0 min group. It has been reported that NGF-induced ERK activation peaks at 5 min after NGF addition and persists for up to 60 min [16]. Phosphorylated and activated proteins do not always remain in a phosphorylated state but are known to undergo transient modifications in stimulus responses such as NGF or other growth factors [27]. Phosphorylation of ERK induced by bLF also exhibited a trend similar to that of growth factors like NGF and is considered a reasonable cellular response. Therefore, it was inferred that bLF activates ERK as rapidly as NGF, but less persistently. Morphological evaluation revealed neurite outgrowth 72 h after treatment with bLF alone, suggesting that it took time for the cellular responses to manifest after ERK activation.

To understand the mechanisms of bLF, we used an ERK pathway inhibitor (PD98059), commonly used for investigating NGF mechanisms [28]. Furthermore, as Trk receptors are upstream of the ERK pathway and TrkA receptors have a high affinity for NGF, we also employed the TrkA receptor inhibitor AG879 [29,30]. The addition of each inhibitor suppressed neurite outgrowth and ERK activity, suggesting that bLF induced neurite outgrowth through the TrkA receptor and ERK pathway, similar to NGF. In contrast, the PI3K/Akt pathway is responsible for NGF-induced neurite elongation in PC12 cells [31]. Although both the ERK and PI3K/Akt pathways are tyrosine kinase receptors, they represent distinct signaling pathways. Additionally, the phosphorylation of Raf by Akt inhibits the activation of the ERK pathway [32]. Although our understanding could be enhanced by examining how bLF acts on the PI3K/Akt pathway and its connection to the ERK pathway, a limitation of this study is that we were unable to examine the involvement of Akt. Therefore, this was a preliminary study on neurite outgrowth, and further studies are required to determine whether bLF treatment affects intracellular organelles and/or the cytoskeleton.

To understand the biological effects of bLF treatment on PC12 cells, mRNA expression levels were determined using quantitative real-time PCR. After 24 h of treatment, we observed significant decreases in the expression of MAP2, which is a differentiation marker involved in neuronal morphogenesis [33], in the 100 and 250 µg/mL bLF-treated groups. In contrast, synaptophysin and synapsin-1 expression levels did not change significantly after 24 h of treatment. Synaptophysin is involved in the regulation of synaptic vesicle endocytosis [34,35], while synapsin-1 is an important gene for fine-tuning synaptic transmission and remodeling [36,37]; both are involved in neurotransmitter release and cytoskeleton organization. Neurite outgrowth was observed 72 h after bLF treatment, and it was predicted that the expression levels of each gene would increase with outgrowth and elongation. However, only MAP2 expression was significantly decreased after 24 h, suggesting that the neurite-outgrowth- and nerve-elongation-inducing effect of LF occurs at the early stage of neuronal morphogenesis, resulting in gene downregulation after 24 h.

## 4. Materials and Methods

### 4.1. Cell Culture and Materials

PC12 cells were purchased from RIKEN BRC (Ibaraki, Japan). Cells were grown in Dulbecco’s modified Eagle’s medium and Ham’s F12 (D-MEM/F-12) medium (Nacalai Tesque, Inc., Kyoto, Japan), including 3151 mg/L D-glucose, 365 mg/L L-glutamine, 3574.5 mg/L 2-[4-(2-hydroxyethyl)-1-piperazinyl]ethanesulfonic acid (HEPES), 55 mg/L sodium pyruvate, containing 10% fetal bovine serum (FBS; #S00EO10002, Biowest Co., Ltd., Nuaillé, France) at 37 °C and 5% CO_2_ in a humidified incubator. bLF obtained from NRL Pharma (Kanagawa, Japan) was dissolved in distilled water and sterilized using a 0.22 µm filter (Millipore Corporation, Billerica, MA, USA). All experiments were performed between passage 10 and 30.

### 4.2. Cell Viability Assay

PC12 cells were seeded at a density of 2.5 × 10^4^ cells/well in 96-well plates. After 24 h of pre-incubation, bLF was added at gradually increasing concentrations (0.01–1000 μg/mL) and incubated for 24 h, and distilled water was added to the control group. Cell viability was evaluated using the cell counting kit-8 (CCK-8; Dojindo Laboratories, Kumamoto, Japan), following the manufacturer’s protocol. Briefly, 10 μL of CCK-8 was added for 1 h at 37 °C and 5% CO_2_ in a humidified incubator. The optical density (OD) was measured at 450 nm using a microplate reader (Infinite F50, TECAN Japan, Co., Ltd., Kawasaki, Japan). All experiments were performed in triplicate and repeated thrice.

### 4.3. Morphological Evaluation of bLF-Induced Neurite Outgrowth

The PC12 cells were seeded at a density of 4 × 10^3^ cells/well in 12-well tissue culture plates coated with type I collagen. After 24 h of pre-incubation, the growth medium was replaced with an FBS-free medium. PC12 cells were treated with bLF at varying concentrations (100, 250, and 500 µg/mL) or NGF (50 ng/mL) as a positive control. Digital images of five random fields were obtained using a phase-contrast inverted microscope (BZ-X800, KEYENECE, Osaka, Japan) equipped with a camera 24 and 72 h after treatment with either bLF or NGF. Morphological evaluations were performed using our previous method [22]. Briefly, neurocyte image analyzer software version 1.5 (Kurabo Industries Ltd., Osaka, Japan) was used to evaluate three parameters: neurite length (length), segment (joint), and branching (passage). The experiments were conducted more than thrice in three wells per group.

### 4.4. Morphological Evaluation Using Inhibitors

To elucidate the mechanisms of action of bLF in neurite outgrowth, AG879, a TrkA receptor inhibitor, and PD98059, a selective MEK inhibitor, were used in the previously described morphological experiments. Briefly, PC12 cells were seeded at a density of 4 × 10^3^ cells/well in 12-well tissue culture plates coated with type-I collagen. After 24 h of pre-incubation, 250 µg/mL of bLF was added in FBS-free medium with or without inhibitors. Digital images of cells were captured using a phase-contrast inverted microscope with a camera 72 h after treatment with either bLF or each inhibitor.

### 4.5. Western Blot Analysis

PC12 cells were seeded at a density of 2 × 10^6^ cells in a 60 mm dish. After 24 h, the cells were treated with 250 µg/mL of bLF in FBS-free medium. Then, 3 mL of tris buffered saline (TBS) was placed in the culture dish and immediately aspirated to remove the medium. The cells were collected at 0 min, 5 min, 10 min, 30 min, 2 h, and 6 h after treatment using 100 μL Pro-Prep (iNtRON Biotechnology, Inc., Seongnam-si, Gyeonggi-do, Republic of Korea). A protease/phosphatase inhibitor cocktail (Cell Signaling Technology, Inc., Danvers, MA, USA) was added to the lysate and subsequently incubated on ice for 15 min. The lysate was centrifuged at 14,000 rpm at 4 °C for 20 min to extract the intracellular proteins. Protein concentrations were determined using the Pierce BCA Protein Assay Kit (Thermo Fisher Scientific, Waltham, MA, USA). Samples (*n* = 4 in each group) containing 10 μg cell protein were prepared as described in our previous study [38]. For the analysis, 12% Mini-PROTEAN TGX Precast Gels (Bio-Rad Laboratories, Hercules, CA, USA) and polyvinylidene fluoride (PVDF) membranes (Millipore Corporation, Billerica, MA, USA) were used. Nonspecific binding was blocked using tris-buffered saline containing 0.1% Tween-20 (TBS-T) and 5% bovine serum albumin (Wako Pure Chemical Industries, Ltd., Osaka, Japan) for 1 h at 25 °C. The membranes were then washed with 0.1% TBS-T (three times, 5 min each) and incubated overnight at 4 °C with primary antibodies against both p44/42 (4695T; Cell Signaling Technology, 1:1000 dilution) and phospho-p44/42 (4370T; Cell Signaling Technology, 1:1000 dilution). Immunoreactive bands were detected using a horseradish peroxidase-conjugated secondary antibody (7074S; Cell Signaling Technology, 1:3000 dilution). The protein bands were visualized using ECL reagents. Images were taken by Amersham Imager 680 (Global Life Science Technologies Japan Co., Ltd., Tokyo, Japan) and analyzed by ImageJ software version 1.51j (freely available from the National Institutes of Health, Bethesda, MD, USA).

### 4.6. Western Blot Analysis Using Various Inhibitors

To understand the molecular mechanisms of action of bLF in neurite outgrowth, the inhibitors AG879 and PD98059 were used in Western blotting experiments. Briefly, PC12 cells were seeded at a density of 2 × 10^6^ cells in a 60 mm dish. After 24 h of pre-incubation, 250 µg/mL of bLF was added in FBS-free medium with or without inhibitors. PD98059 was pre-incubated for 2 h followed by the addition of bLF. AG879 was simultaneously administered with bLF. For both experiments, the cells were collected 5 min after the bLF treatment.

### 4.7. RNA Isolation and Real-Time Quantitative PCR (RT-qPCR)

PC12 cells were seeded at a density of 5 × 10^5^ cells/60 mm dish and cultured in DMEM containing 10% FBS. After incubation for 24 h, the growth medium was replaced with FBS-free media, and 250 μg/mL or 500 μg/mL of bLF along with 50 ng/mL of NGF were added for 24 h. Total RNA was extracted using Isogen (Nippon Gene, Tokyo, Japan), and chloroform (Nacalai Tesque Inc., Kyoto, Japan) was added to the collected samples. Isopropanol (Nacalai Tesque, Inc.) was added to the supernatant, and the precipitate was collected via centrifugation at 14,000 rpm at 4 °C for 15 min. The precipitate was then resuspended in sterile water, and the RNA concentration was measured. Total RNA template was adjusted to 1 ng/μL, and cDNA was synthesized using ReverTra Ace qPCR RT Master Mix with gDNA Remover (Toyobo Co., Osaka, Japan), following the instructions provided by the manufacturer. Quantitative real-time PCR was performed using THUNDERBIRD SYBR qPCR Mix (Toyobo Co., Osaka, Japan). Synapsin-1, synaptophysin, and MAP2 mRNA expression levels were assessed using a LightCycler 96 system (Roche Diagnostics K.K., Tokyo, Japan). The reaction condition of a three-step real-time PCR amplification program comprised an initial denaturation step at 95 °C for 30 s, followed by an amplification step of 50 denaturation cycles at 95 °C for 5 s, primer annealing at 60 °C for 30 s, extension at 72 °C for 60 s and plate read, and a melting curve step from 55 to 95 °C with 0.5 °C increments for 5 s, followed by a plate read. Their relative expression was normalized to that of the housekeeping gene GAPDH. Oligonucleotide primers for amplifying related genes were designed and checked using Primer-BLAST (NCBI, NIH, MD, USA), as listed in Table 1.

### 4.8. Statistical Analysis

Data were expressed as the mean ± standard deviation (SD). Comparisons between the various treatment groups and the corresponding controls in each experiment were performed using one-way analysis of variance (ANOVA), followed by Tukey’s multiple comparison test. Statistical analyses were performed using SPSS software version 28.0 (IBM Corp., Armonk, NY, USA). Differences were considered statistically significant at probability (*p*) values < 0.05.

## 5. Conclusions

The present study revealed that LF promotes neurite elongation through the activation of the ERK pathway via the TrkA receptor, similar to NGF. Therefore, bLF could lead to the development of new treatment approaches for neurodegenerative diseases, such as Alzheimer’s disease, Parkinson’s disease, and depression.

## Figures and Tables

**Table 1:** Specific primer sequences used for real-time PCR.

Genes	Primer Sequences (5′–3′)
GAPDH Forward	AAGTTCAACGGCACAGTCAA
GAPDH Reverse	GATCTCGCTCCTGGAAGATG
Synapsin-1 Forward	CCCAGATGGTTCGACTACAC
Synapsin-1 Reverse	GGGTATGTTGTGCTGCTGAG
Synaptophysin Forward	AAAGGCCTGTCCGATGTGAAG
Synaptophysin Reverse	TCCCTCAGTTCCTTGCATGTG
MAP2 Forward	GATCAACGGAGAGCTGACCT
MAP2 Reverse	TTGGGCCTCCTTCTCTTGTT

## Data Availability

Data will be made available on request.
